# Резистентный к радиойодтерапии дифференцированный рак щитовидной железы у детей и подростков. Ретроспективное исследование

**DOI:** 10.14341/probl13649

**Published:** 2026-07-22

**Authors:** К. Ю. Слащук, М. В. Рейнберг, П. О. Румянцев, П. А. Никифорович, А. П. Кононыхина, А. П. Першина-Милютина, А. В. Аредов, М. С. Шеремета, М. В. Дегтярев, А. А. Трухин, О. А. Чикулаева, Е. В. Нагаева, Д. Н. Бровин, Р. А. Черников, О. Б. Безлепкина, В. А. Петеркова, Н. Г. Мокрышева, И. И. Дедов

**Affiliations:** Национальный медицинский исследовательский центр эндокринологии им. академика И.И. Дедова Россия; National Medical Research Center for Endocrinology named after Academician I.I. Dedov Russian Federation; Мой Медицинский Центр «Высокие технологии» Россия; My Medical Center High Technologies Russian Federation; Национальный медицинский исследовательский центр нейрохирургии им. академика Н.Н. Бурденко Россия; N.N. Burdenko National Medical Research Center for Neurosurgery Russian Federation; Санкт-Петербургский государственный университет» Клиника высоких медицинских технологий имени Н.И. Пирогова Россия; Saint Petersburg State University Russian Federation

**Keywords:** дифференцированный рак щитовидной железы, дети, подростки, терапия радиоактивным йодом, резистентность к радиойодтерапии, таргетная терапия, differentiated thyroid carcinoma, children, adolescents, radioiodine therapy, radioiodine­refractory disease, targeted therapy

## Abstract

**ВВЕДЕНИЕ:**

ВВЕДЕНИЕ. Дифференцированный рак щитовидной железы (ДРЩЖ) у детей — редкое заболевание, характеризующееся склонностью к регионарному и отдаленному метастазированию, однако при этом имеющее благоприятный долгосрочный прогноз в большинстве случаев. Радиойодрезистентные (РЙР) формы заболевания представляют собой особую клиническую проблему, которая может составлять 10–30% всех случаев ДРЩЖ и требует углубленного изучения клинического течения, факторов прогноза и возможностей терапии данной подгруппы. Цель данного исследования — комплексная оценка исходов лечения у детей, получивших комбинированное лечение по поводу ДРЩЖ (операция и радиойодтерапия (РЙТ)), с акцентом на распространенные и РЙР формы заболевания.

**МАТЕРИАЛЫ И МЕТОДЫ:**

МАТЕРИАЛЫ И МЕТОДЫ. Ретроспективно проанализированы медицинские данные 278 пациентов в возрасте от 5 до 18 лет, перенесших первичное хирургическое лечение в период с 2008 по 2022 гг. и получивших один или несколько курсов РЙТ в ФГБУ «НМИЦ эндокринологии им. академика И.И. Дедова» Минздрава России с декабря 2015 по март 2024 гг. В исследование включены пациенты с распространенным заболеванием (высокий риск рецидива на момент диагностики) и соответствующие одному из критериев РЙР, с медианой наблюдения 48,0 месяцев [21,5; 62,0].

**РЕЗУЛЬТАТЫ:**

РЕЗУЛЬТАТЫ. Из 278 пациентов у 39 (14%) выявлено распространенное заболевание, среди них 4 пациента достигли ремиссии, 29 — стабилизации, 6 — биохимического/структурного прогрессирования. Выживаемость без прогрессирования в группе РЙР пациентов составила 85%, 5-летняя общая выживаемость — 100%.

**ЗАКЛЮЧЕНИЕ:**

ЗАКЛЮЧЕНИЕ. По результатам исследования предложена новая классификация, основанная на исходной способности метастазов накапливать 131I и динамической оценке ответа на лечение после РЙТ (прогрессирование или стабилизация). Эта система направлена на оптимизацию алгоритмов лечения и наблюдения. Стратегия ведения детей с РЙР ДРЩЖ должна быть сбалансированной: с одной стороны — избегать избыточной терапии при стабильном течении заболевания, с другой — своевременно применять современные системные подходы при наличии факторов риска прогрессирования заболевания.

## Abstract (en)

**BACKGROUND:**

BACKGROUND: Differentiated thyroid carcinoma (DTC) in children is a rare malignancy characterized by a high propensity for regional and distant metastases yet generally associated with a favorable long-term prognosis. Radioiodine-refractory (RAIR) disease represents a distinct clinical challenge, accounting for approximately 10–30% of pediatric DTC cases, and warrants comprehensive investigation of its clinical course, prognostic factors, and therapeutic options. This study aimed to perform an integrated assessment of treatment outcomes in children undergoing combined therapy for DTC (surgery and radioiodine therapy, RAI), with a particular focus on advanced and RAIR disease.

**MATERIALS AND METHODS:**

MATERIALS AND METHODS: We retrospectively analyzed medical records of 278 patients aged 5–18 years who underwent primary surgical treatment between 2008 and 2022, followed by one or more courses of RAI at the Endocrinology Research Centre (Moscow, Russia) from December 2015 to March 2024. The study included patients with advanced disease (high risk of recurrence at diagnosis) fulfilling at least one RAIR criterion, with a median follow-up of 48.0 months [21.5; 62.0].

**RESULTS:**

RESULTS: Among 278 patients, 39 (14%) were diagnosed with advanced disease. Of these, 4 achieved remissions, 29 had stable disease, and 6 experienced biochemical and/or structural progression. Progression-free survival in the RAIR cohort was 85%, while the 5-year overall survival reached 100%.

**CONCLUSION:**

CONCLUSION: Based on study findings, we propose a novel classification system integrating both the baseline ability of metastases to accumulate ¹³¹I and the dynamic response to RAI (progression vs. stabilization). This framework is designed to optimize treatment and follow-up algorithms. The management of RAIR pediatric DTC should remain balanced: avoiding overtreatment in stable disease, while ensuring timely initiation of modern systemic therapies in patients with risk factors for progression.

## Обоснование

ДРЩЖ у детей и подростков встречается значительно реже чем у взрослых, но является одной из наиболее частых солидных опухолей в педиатрической популяции. Распространенность ДРЩЖ в структуре всех злокачественных опухолей может составлять до 3% от общего количества и до 15% злокачественных солидных опухолей головы и шеи в детском возрасте [1]. По данным на 2021 год, в России абсолютное число заболеваемости среди детей в возрасте 0–4, 5–9, 10–14 и 15–19 лет составляет 2, 8, 49, 138 случаев соответственно [2]. Чаще заболевают девочки (соотношение мальчик/девочка 1:5) [1][2][14].

Наиболее распространенной гистологической формой у детей является папиллярный рак щитовидной железы (ПРЩЖ), на долю которого приходится 70–99% всех случаев, в то время как фолликулярный рак (ФРЩЖ) встречается значительно реже [3]. Как правило, оба варианта демонстрируют более благоприятное течение по сравнению с аналогичными опухолями у взрослых, несмотря на более высокий процент регионарного и отдаленного метастазирования на момент постановки диагноза. Среди генетических изменений при ДРЩЖ выявляются соматические перестройки генов RET/PTC, онкогенные слияния NTRK и точечные мутации в BRAF [4–6].

Однако несмотря на часто распространенные формы заболевания, нередко сопровождающегося множественным поражением лимфатических узлов шеи, и высокий процент отдаленных метастазов (чаще в легкие — мелкоочаговой, микронодулярной формы, до 30% случаев), общий прогноз у таких детей остается хорошим, ожидаемая 10-летняя выживаемость при любой распространенности заболевания превышает 95–98% [7–10]. Смертность в России на 2021 год составила 0% [2]. Считается, что ДРЩЖ в детском возрасте возникает спорадически из-за соматических генетических изменений, которые приводят к онкогенезу, главным образом изменяя сигнальные пути, активируемые митоген-активируемой протеинкиназой и фосфоинозитол-3-киназой [11]. Ряд авторов отмечает более высокую чувствительность данной когорты пациентов к лечению радиоактивным йодом, что как правило должно приводить к полному терапевтическому ответу отдаленных метастазов после одного или нескольких курсов терапии радиоактивным йодом [9][12][13]. Таким образом, считается, что агрессивные случаи ДРЩЖ у детей, которые требуют системной терапии, помимо стандартного применения радиоактивного йода и супрессивной гормональной терапии, выявляются редко.

Клинические рекомендации по диагностике, ведению и оценке клинического риска рецидива ДРЩЖ в педиатрической популяции были основаны на клинических руководствах для взрослых. Недостаток информации об эффективности терапии у детей требует выделения специфических групп пациентов и углубленного анализа отдаленных результатов лечения и наблюдения. Актуальными вопросами остаются: определение оптимального объема хирургического лечения, показания и расчет активности ¹³¹I, сроки проведения РЙТ, а также уточнение критериев РЙР, которые по-прежнему четко не определены, периодичность динамического наблюдения и обоснование целесообразности системной терапии у пациентов с длительно стабилизированным течением болезни [14].

Многие международные гайдлайны рекомендуют проведение адъювантной РЙТ ¹³¹I при высокой и, избирательно, в промежуточной послеоперационной группе риска рецидива ДРЩЖ с целью аблации остаточной тиреоидной ткани, а также для выявления и лечения отдаленных метастазов. Терапевтические цели данного лечения заключаются в достижении биохимической ремиссии (неопределяемого уровня тиреоглоубилна (ТГ) и антител к тиреоглоубилну (АТ к ТГ) в крови), а также структурной ремиссии (отсутствия визуализируемых очагов опухоли) [14]. Стандартно, с целью лечебного воздействия на очаги, захватывающие ¹³¹I, могут проводиться повторные курсы РЙТ с интервалами 3–12 месяцев, до полного исчезновения очагов накопления ¹³¹I и достижения неопределяемого или стабильно-низкого уровня ТГ в сыворотке крови.

По данным разных авторов в 10–30% случаев ДРЩЖ в педиатрической группе, получавших терапию ¹³¹I, демонстрируются неполный терапевтический эффект РЙТ, что позволяет отнести их к категории РЙР пациентов [14][15]. Молекулярные механизмы резистентности к РЙТ связаны с генетическими и эпигенетическими изменениями, нарушающими работу натрий-йодидного симпортера (НЙС, кодируется геном SLC5A5), вероятно, включая: снижение/потерю экспрессии НЙС, аномальный посттрансляционный процессинг, нарушение внутриклеточного транспорта.

Благодаря достижениям в области молекулярной генетики и таргетной терапии появились новые возможности для пациентов с резистентным к РЙТ раком щитовидной железы. Пациентам с ДРЩЖ с наличием йод-негативных метастазов, которым невозможно провести хирургическое лечение, может быть назначена таргетная терапии препаратами из группы тирозинкиназных ингибиторов (ТКИ). Говоря о молекулярно-генетическом ландшафте опухолей, в серии исследований de Sousa и соавт., генетические альтерации были выявлены в 85% случаев ДРЩЖ у детей [16]. Наиболее частыми были мутации RET, NTRK1, NTRK3, реже — ALK, MET. В отличие от взрослых, BRAF-мутация встречается реже [16][17]. До сих пор неоднозначным является вопрос о том, является ли наличие мутации в гене DICER1 предиктором развития РЩЖ у детей. По некоторым данным, при фолликулярных опухолях данная мутация наиболее распространена [18][19], но также встречается с определенной частотой в ПРЩЖ, низкодифференцированных карциномах [20][21] и доброкачественных новообразованиях [20].

Препаратами, зарегистрированными в России для лечения прогрессирующего, распространенного ДРЩЖ резистентного к РЙТ у взрослых пациентов, являются Сорафениб и Ленватиниб.

Сорафениб является первым одобренным в мире ТКИ, на основании исследования DECISION, для лечения взрослых пациентов с местнораспространенным или метастатическим РЙР ДРЩЖ. В многоцентровом, рандомизированном, двойном слепом клиническом исследовании 3 фазы было показано увеличение выживаемости без прогрессирования (ВБП) в группе Сорафениба почти в 2 раза по сравнению с плацебо [22]. Сорафениб подавляет на молекулярном уровне активность BRAF и CRAF, что ведет к снижению сигнальной активности по каскаду MAPK (RAF–MEK–ERK). Он также ингибирует рецепторы VEGFR-1/2/3, PDGFR-β, c-KIT и в меньшей степени — RET, воздействуя как на пролиферацию, так и на ангиогенез опухоли.

Ленватиниб — это селективный ингибитор рецепторных тирозинкиназ, действующий на множественные мишени (VEGFR-1–3, FGFR-1–4, PDGFR-α, c-KIT и неселективно RET), что также обуславливает противоопухолевую и антиангиогенную активность. Препарат одобрен FDA для лечения взрослых с местнораспространенным или метастатическим, прогрессирующим РЙР ДРЩЖ. В 2018 г. впервые опубликовано наблюдение успешного лечения Ленватинибом 3 детей с РЙР ДРЩЖ и метастатическим поражением легких. У двоих из трех пациентов отмечалась стабилизация заболевания через 23 месяца и 11 месяцев после начала терапии соответственно. В третьем случае было обнаружено слияние гена NTRK и пациент был переведен на терапию NTRK-ингибитором [23]. Ленватиниб также продемонстрировал эффективность у пациентов с прогрессированием заболевания после терапии другим ингибиторами тирозинкиназ, возможно, потому что ингибировал сразу нескольких путей, участвующих в ангиогенезе и опухолевой пролиферации. Таким образом, применение Ленватиниба можно рассматривать в качестве опции лечения педиатрических пациентов с прогрессирующим РЙР ДРЩЖ.

Доказательная база в отношении способности Сорафениба и Ленватиниба усиливать захват ¹³¹I остается ограниченной, и в настоящее время такие эффекты не подтверждены.

Учитывая гетерогенную мутационную характеристику ДРЩЖ у педиатрических пациентов, при прогрессирующем течении заболевания возможно рассмотреть и опухоль-специфичную, агностическую таргетную терапию при верифицированных мутациях (например, NTRK, RET, BRAF, MEK): вемурафениб/дабрафениб [24], селуметиниб [25], ларотректиниб [26], энтректиниб [27], селперкантиниб [28], пралсетиниб [29].

Агностическая терапия в онкологии — это подход к лечению рака, при котором выбор терапии определяется не локализацией опухоли, а наличием определенных мутаций в опухолевых клетках.

Ларотректиниб является ингибитором тропомиозинкиназ с ингибированием RAF-MEK-ERK или PI3K-AKT сигнальных путей, показал единичных клинических случаях высокую эффективность.

Селуметиниб (ингибитор MAPK-киназы MEK 1, 2) восстанавливает чувствительность к РЙТ у пациентов, у которых раннее опухоль не поглощала ¹³¹I. Предварительное лечение Селуметинибом увеличивало захват йода у 12 из 20 пациентов (у 4 из 9 пациентов с мутациями BRAF и у 5 из 5 пациентов с мутациями NRAS) [25].

Вемурафениб (ингибитор BRAF) применялся у 17 взрослых пациентов с РЙР ДРЩЖ. Частичный ответ наблюдался у 47% пациентов, стабилизация заболевания — более чем у 50% [24]. Полный перечень препаратов, используемых для таргетной терапии ДРЩЖ в детском возрасте, перечислены в таблице 1.

**Table:** Таблица 1. Агностическая таргетная терапия ДРЩЖ в мировой практике, включая детскую группу *Прошедшие комбинированное лечение (таргетная терапия + радиойодтерапия в контексте редифференцировки опухоли)ПО — полный ответЧО — частичный ответС — стабилизацияП — прогрессирование

Исследование	Препарат	Точка приложения	Кол-во детей в исследовании	% ответа на терапию	Показания к применению, возраст
Steven GW и др., 2022 [29]	Ларотректиниб	NTRK 1; NTRK 3	2	ПО — 1ЧО — 1	при NTRK fusion-мутациях, для детей с 1 месяца
Desai AV и др., 2022 [27]	Энтректиниб	ROS1 fusionNTRK 1/2/3ALK fusion	43 (26 были доступны для оценки исходов)	ПО — 7 (26,9%)ЧО — 8 (30,8%)С — 7 (26,9%)П — 2 (7,7%)	при NTRK fusion-мутациях, для детей с 12 лет
Wirth LJ и др., 2020 [28]	Селперкантиниб	RET	162 (общая выборка, включая детей)	ПО — 16 (10%)ЧО — 101 (62,3%)С — 38 (23,5%)П — 3(1,9%)	при RET fusion-мутациях, для детей с 12 лет
Vivek Subbiah и др., 2021 [29]	Пралсетиниб	RET	N (единичные случаи применения у детей)	Ответ на терапию получен у 56 — 91% пациентов	при RET fusion-мутациях, для детей с 12 лет
Mahajan P и др., 2018 [23]	Ленватиниб	VEGFR-1–3, FGFR-1–4, PDGFR-α, c-KIT, RET	3	ЧО — 2С — 1	для детей с 18 лет
Alan L. Ho и др., 2013 [25]	Селуметиниб	BRAFV600, NRAS	8*	ЧО — 5С — 3	для детей с 2-х лет
Mark W Kiera и др. [30]	Дабрафениб	BRAF V600E	27	ЧО — 14П — 10С — 1	при BRAF мутации, для детей с 6 лет

Исходя из вышеописанных данных, ТКИ способны приводить к достижению клинического эффекта и рентгенологического ответа опухоли на терапию, а также «редифференцировать» опухоль, потенциально восстанавливая/улучшая способность накапливать ¹³¹I. Однако требуется больше исследований для оценки эффективности и безопасности применения ТКИ у детей, а также анализ отдаленных результатов лечения. Так как системная терапия ассоциирована с риском развития различных нежелательных явлений, ее применение не должно рассматриваться у пациентов с резистентностью к РЙТ, но стабильным, или медленно-прогрессирующим течением заболевания.

## Цель ИССЛЕДОВАНИЯ

Оценить результаты динамического наблюдения и персонализировать подходы к ведению пациентов с резистентным к радиойодтерапии ДРЩЖ после комбинированного лечения в педиатрической группе на базе одного центра — на базе одного центра — ФГБУ «НМИЦ эндокринологии им. академика И.И. Дедова» Минздрава России.

## МАТЕРИАЛЫ И МЕТОДЫ

Ретроспективно проанализированы медицинские данные 278 пациентов младше 18 лет с ДРЩЖ, перенесших хирургическое лечение в период с 2008 по 2022 гг. и получивших один или несколько курсов РЙТ в ФГБУ «НМИЦ эндокринологии им. академика И.И. Дедова» Минздрава России с декабря 2015 по март 2024 гг.

Всем пациентам была выполнена тиреоидэктомия с различным объемом удаления лимфатических узлов шеи. В послеоперационном периоде пациенты получали супрессивную терапию левотироксином натрия (ЛТ4) с контролем уровня ТТГ и свободного Т4 (свТ4) (целевой уровень ТТГ<0,1 мМЕ/л, свТ4 в пределах референсного интервала лаборатории).

Во всех случаях метастазы в легкие были подтверждены клинически на основании сцинтиграфии всего тела и данных компьютерной томографии, с морфологической верификацией очагов в двух случаях.

Стадирование по системе pTNM основывалось на 8-м издании классификации Американского объединенного комитета по раку (AJCC 8th edition) [31].

Патоморфологический диагноз соответствовал 4-му изданию классификации ВОЗ [32].

Определение резистентности к радиойодтерапии проводилось по следующим критериям:

Обследования, включая лабораторные (ТТГ, свТ4, ТГ, АТ к ТГ) и инструментальные методы (УЗИ ложа щитовидной железы и лимфатических узлов шеи, КТ органов грудной клетки; по показаниям — диагностическая сцинтиграфия всего тела (дСВТ) с 123I или 131I, ПЭТ/КТ с 18F-фтордезоксиглюкозой), выполнялись каждые 3–6 месяцев.

В ходе анализа данных пациенты дополнительно были разделены на 2 клинические группы: с ¹³¹I-позитивными отдаленными метастазами и ¹³¹I-негативными, при этом критерием включения в последнюю группу было отсутствие накопление ¹³¹I на посттерапевтических диагностических снимках после однократно проведенной радиойодтерапии, при наличии клинических данных за отдаленное метастазирование

## Статистический анализ

Статистический анализ проведен с использованием языка программирования Python (v. 3.11) и программного пакета Statistica v.13 (TIBCO Inc., США). Описательная статистика количественных показателей представлена медианами, 1-м и 3-м квартилями в виде Me [ Q1; Q3], качественных показателей — абсолютными и относительными частотами в виде n (%). Сравнение трех независимых групп для количественных данных выполнялось с помощью критерия Краскела-Уоллиса, а качественных — при помощи критерия Фримена-Холтона. Был проведен корреляционный анализ при помощи критерия Фримена-Холтона и post-hoc анализа. Анализ выживаемости был выполнен с помощью логарифмического рангового теста и построения графиков Каплана-Мейера. Сравнение двух независимых групп для количественных данных выполнялось с помощью критерия Манна–Уитни. Сравнение двух независимых групп для качественных признаков выполнялось с помощью критерия Фримена-Холтона и точного двустороннего критерия Фишера, в случае бинарных признаков. Критический уровень статистической значимости при проверке статистических гипотез принят равным 0,05. При множественных сравнениях применялась поправка Бонферрони путем коррекции критического уровня значимости. Рассчитанные уровни значимости в диапазоне от критического до 0,05 описаны в качестве индикаторов статистической тенденции.

## Этическая экспертиза

Исследование одобрено локальным этическим комитетом Федерального государственного бюджетного учреждения «Национальный медицинский исследовательский центр эндокринологии им. академика И.И. Дедова» Министерства здравоохранения Российской Федерации; выписка из протокола №19 заседания локального этического комитета от 3.10.2025г. Заключение: научная работа соответствует этическим стандартам добросовестной клинической практики.

## Результаты

Все 39 пациентов (что составило 14% от всех пролеченных больных) относились к группе высокого послеоперационного риска рецидива (согласно АТА 2015) и получили от 1 до 15 курсов РЙТ. Суммарная активность введенного ¹³¹I варьировала от 2,32 до 42,8 ГБк. После проведенного курса РЙТ всем пациентам выполнялась посттерапевтическая сцинтиграфия всего тела (птСВТ) и ОФЭКТ/КТ, а опухоли классифицировались как резистентные к РЙТ согласно указанным критериям. Полная характеристика пациентов представлена в таблице 2.

**Table:** Таблица 2. Характеристика исследуемой группы пациентов, наблюдавшихся в период с 2015 по 2024 гг. ТЭ — тиреоидэктомияЦЛАЭ — центральная лимфаденэктомияБЛАЭ — боковая лимфаденэктомия (БЛАЭ1 — с одной стороны; БЛАЭ2 — с двух сторон)СЛАЭ — селективная лимфаденэктомия (повторная)R2 — остаточная опухоль (не радикальная операция)л/у — лимфатические узлы

№	Возраст на момент диагноза	Пол	Объем операции	Кол-во РЙТ	Суммарная активность, ГБк	Гистологичесий тип ПРЩЖ	pTNM	Локализация метастазов
1	16	м	ТЭ+БЛАЭ1+ЦЛАЭ	3	10,3	классический	T4aN1bM1	легкие, л/у
2	17	м	ТЭ+БЛАЭ1+ЦЛАЭ	5	23,2	фолликулярный вариант	T2N1aM1	легкие, л/у
3	12	ж	ТЭ+БЛАЭ1	7	42,8	классический	T3bN1bM1	легкие
4	10	м	ТЭ+БЛАЭ1	5	22,5	фолликулярный вариант	T3bN1bM1	легкие
5	13	м	ТЭ+БЛАЭ1	2	6,2	диффузно-склерозирующий	T3bN1bM1	легкие
6	5	ж	ТЭ+ЦЛАЭ+БЛАЭ2	3	5,07	классический + фолликулярный варианты	T4aN1bM1	легкие
7	6	ж	ТЭ+ЦЛАЭ+БЛАЭ2	3	9,75	фолликулярный вариант	T4aN1bM1	легкие
8	10	ж	ТЭ+ЦЛАЭ	3	12,4	широкоинвазивный, светлоклеточный	Т3bN1aM1	легкие
9	7	м	ТЭ+БЛАЭ1+ЦЛАЭ+СЛАЭ	3	14,68	классический	T4aN1bM1	легкие
10	16	ж	ТЭ+ЦЛАЭ+БЛАЭ1	2	6,5	фолликулярный вариант	T2N1aM1	легкие
11	14	ж	ТЭ	2	7,5	фолликулярный вариант	T2N0M1	легкие
12	14	ж	ТЭ+ЦЛАЭ+БЛАЭ1	3	14	классический	T3bN1aM1	легкие
13	13	ж	ТЭ+ЦЛАЭ+БЛАЭ2	2	8,9	классический	T3bN1bM1	легкие, л/у
14	8	ж	ТЭ+ЦЛАЭ+БЛАЭ2	15	35,5	фолликулярный вариант	T4aN1bM1	легкие
15	11	ж	ТЭ+ЦЛАЭ+БЛАЭ2	11	30	классический	T4аN1bM1	легкие
16	7	ж	ТЭ+ЦЛАЭ+БЛАЭ2	15	33,3	фолликулярный вариант	T3bN1bM1	легкие
17	14	ж	ТЭ	1	4,4	классический	T2NxM1	легкие
18	10	ж	ТЭ+ЦЛАЭ+БЛАЭ2	4	20,2	классический	T4bN1bM1	легкие
19	11	м	ТЭ+БЛАЭ1	6	29,18	классический	T3bN1bM1	легкие
20	9	ж	ТЭ+ЦЛАЭ+БЛАЭ2	2	9,2	классический	T3bN1bM1	легкие
21	6	м	ТЭ+ЦЛАЭ+БЛАЭ2	2	2,32	классический	T3aN1bM1	легкие
22	15	ж	ТЭ+ЦЛАЭ+БЛАЭ2+СЛАЭ	1	5,58	классический	T4aN1bN1	легкие
23	13	ж	ТЭ+ЦЛАЭ	2	9,28	фолликулярный вариант	T3bN1aM1	легкие
24	11	ж	ТЭ+ЦЛАЭ+БЛАЭ2	1	3,5	классический	T1bN1bM1	легкие
25	12	м	ТЭ+БЛАЭ2	2	9,6	классический	T2(m)N1bM1	легкие, л/у
26	11	ж	ТЭ+ЦЛАЭ+БЛАЭ2	4	19,62	фолликулярный вариант	T2N1bM1	легкие
27	9	м	ТЭ+ЦЛАЭ+БЛАЭ2	1	1,91	фолликулярный вариант	T1bN1bM1	легкие
28	6	м	ТЭ+ЦЛАЭ+БЛАЭ2	5	13,4	классический	T3b(m)N1bM1	легкие
29	12	м	ТЭ+ЦЛАЭ+БЛАЭ2	2	6,2	классический	T1b(m)N1bM1	легкие
30	16	ж	ТЭ+ЦЛАЭ+БЛАЭ2	3	15,72	классический	T1b(m)N1bM1	легкие, печень
31	17	ж	ТЭ+ЦЛАЭ+БЛАЭ1	1	4,2	классический	T2N1bM1	легкие
32	15	ж	ТЭ+БЛАЭ+ЦЛАЭ	4	19,2	классический	pT4aN1bM1	легкие
33	5	ж	ТЭ+ БЛАЭ+ЦЛАЭ	3	5,17	классический	T2N1bM1	легкие
34	16	м	ТЭ+ БЛАЭ+ЦЛАЭ	2	9,43	высококлеточный	Т1bN1bcM1	легкие
35	17	м	ТЭ+БЛАЭ+ЦЛАЭ	2	9,13	фолликулярный вариант	T1bN1bM1	легкие
36	8	м	ТЭ+БЛАЭ+ЦЛАЭ	3	6,35	классический	T3bN1bM1	легкие
37	7	м	ТЭ+БЛАЭ+ЦЛАЭ	2	6,5	классический	T3b(m)N1bM1	легкие
38	8	м	ТЭ+БЛАЭ+ЦЛАЭ	2	3,77	фолликулярный вариант	T4aN1bM0	R2
39	12	ж	ТЭ+БЛАЭ+ЦЛАЭ	1	3,74	фолликулярный вариант	T4bN1bM1	легкие, R2

Длительность наблюдения составила 48 месяцев [ 21,5; 62,0] (4,0 года [ 1,79; 5,17]).

В структуре 39 пациентов: 16 мальчиков, 23 девочки, Me [ Q1; Q3] — 11 [ 8, 14]. Из них у 4 пациентов (10%) со временем была достигнута полная биохимическая и структурная ремиссия, у 29 пациентов (74%) — стабилизация, и 6 пациентов (15%) прогрессировали. В одном случае из шести наблюдалось агрессивное течение ДРЩЖ у пациентки 8 лет (с положительной мутацией BRAF V600E) — в виде рентгенологического прогрессирования и роста уровня ТГ. Несмотря на высокодозную РЙТ, отмечался непрерывный рост метастатических очагов в легких, что послужило основанием для назначения Ленватиниба. На фоне терапии был достигнут частичный ответ, сохраняющийся все время наблюдения. Выживаемость без прогрессирования составила 85% при общей 5-летней выживаемости 100%. Медиана безрецидивной выживаемости составила 54 месяца.

Медиана наблюдения за пациентами до прогрессирования составила 29,5 месяца, что акцентирует внимание на целесообразности активного наблюдения в течение первых 2–3 лет с момента постановки диагноза. По истечении данного периода наблюдения большинство пациентов оставались стабильными и демонстрировали благоприятный ответ на лечение. По истечении медианы в 58 месяцев наблюдения повторных случаев прогрессирования и/или рецидива не наблюдалось в нашей выборке, что позволяет сделать вывод о возможности снижения интенсивности динамического наблюдения пациентов педиатрической группы спустя 4–5 лет (рис. 1). Взаимосвязь исходов заболевания и средней продолжительности наблюдения указана в таблице 3.

**Figure:**
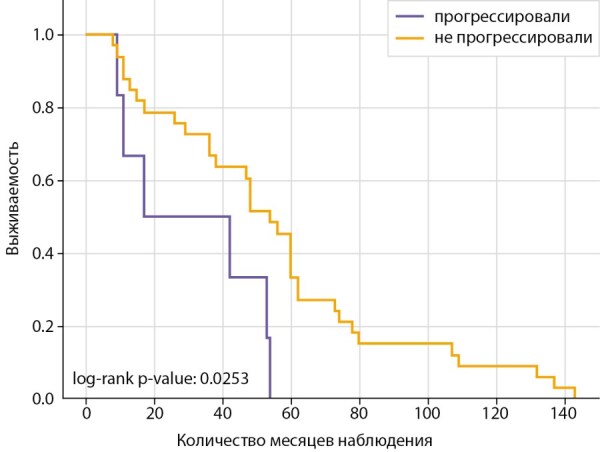
Рисунок 1. График сравнения продолжительности наблюдения пациентов в группах без прогрессирования и с прогрессированием заболевания.

**Table:** Таблица 3. Взаимосвязь исходов заболевания (прогрессирование/стабилизация/ремиссия) и средней продолжительности наблюдения пациентов, с коррекцией на пол и возраст ¹ — критерий Фримена-Холтона² — критерий Краскела-УоллисаПоправка Бонферрони: p0=0,05/4=0,0125

Признак	Стабилизация	Ремиссия	Прогрессирование	p
N	Me [ Q1; Q3]/ n (%)	N	Me [ Q1; Q3]/ n (%)	N	Me [ Q1; Q3]/ n (%)
Пол	М	29	13 (45%)	4	0 (0%)	6	2 (33%)	0,271¹
Ж	16 (55%)	4 (100%)	4 (67%)
Возраст 1	младше 14 лет	29	22 (76%)	4	1 (25%)	6	2 (33%)	0,033¹
14 лет и старше	7 (24%)	3 (75%)	4 (66%)
Возраст 2	младше 8 лет	29	5 (17%)	4	1 (25%)	6	2 (33%)	0,102¹
от 8 до 14 лет	17 (59%)	0 (0%)	2 (33%)
14 лет и старше	7 (24%)	3 (75%)	2 (33%)
Продолжительность наблюдения (месяцы)	29	48 [ 26; 73]	4	58 [ 52; 65]	6	29,5 [ 12,5; 50,3]	0,168²

Пациенты с прогрессированием и стабилизацией/ремиссией не различались по возрасту и полу. Размер первичной опухоли, количество и величина метастазов, гистологический тип, стадия TNM и суммарная активность ¹³¹I не влияли на исход заболевания.

Тиреоглобулин как высокочувствительный онкомаркер в нашем исследовании достоверно коррелировал с исходом заболевания (уровень стимулированного ТГ колебался от 4,59 до 21310 нг/мл):

**Table:** Таблица 4. Взаимосвязь уровня ТГ (повышение/снижение/стабилизация) и исходов заболевания (прогрессирование/стабилизация/ремиссия) ¹ — критерий Фримена-Холтона² — ТКФ (Точный критерий Фишера)

Признак	Снижение	Без изменений	Увеличение	p
N	n (%)	N	n (%)	N	n (%)
Прогрессирование	да	23	0 (0%)	14	4 (28,6%)	2	2 (100%)	p1-2=0,016²
нет	23 (100%)	10 (71,4%)	0 (0%)
Стабилизация	23	19 (82,6%)	10	10 (100%)	0	-	p1-2=0,289²
Ремиссия	4 (17,4%)	0 (0%)	-
Прогрессирование	23	0 (0%)	14	4 (29%)	2	2 (100%)	p1-2=0,040¹
Стабилизация	19 (83%)	10 (71%)	0 (0%)
Ремиссия	4 (17%)	0 (0%)	0 (0%)

В процессе наблюдения у 29 пациентов уровень ТГ оставался стабильно повышенными без значимой тенденции к росту, по данным дСВТ накопления ¹³¹I в ранее выявленных очагах не отмечалось, отсутствовала отрицательная динамика в плане увеличения их размеров по данным КТ (табл. 5).

**Table:** Таблица 5. Взаимосвязь уровня ТГ (повышение/снижение/стабилизация) и исходов заболевания (прогрессирование/стабилизация/ремиссия) ¹ — критерий Фримена-Холтона² — ТКФ (точный критерий Фишера)

Признак	Снижение	Без изменений	Увеличение	p, критерий Фримена-Холтона
N	n (%)	N	n (%)	N	n (%)
Прогрессирование заболевания	да	5	0 (0%)	32	4 (12,5%)	2	2 (100%)	p1-2=0,820²
нет	5 (100%)	28 (87,5%)	0 (0%)
Стабилизация	5	5 (100%)	28	24 (85,7%)	0	-	p1-2=0,744²
Ремиссия	0 (0%)	4 (14,3%)	-
Прогрессирование	5	0 (0%)	32	4 (13%)	2	2 (100%)	p1-2=0,907¹
Стабилизация	5 (100%)	24 (74%)	0 (0%)
Ремиссия	0 (0%)	4 (13%)	0 (0%)

Изменения уровня АТ к ТГ на нашей выборке пациентов не коррелировали с исходом заболевания. Всего в исследовании было 11 пациентов с повышенным уровнем АТ к ТГ, исходно от 3-кратного до 75-кратного повышения относительно верхней границы референсного интервала. У 6 пациентов АТ к ТГ снизились в процессе динамического наблюдения, но полностью нормализовались лишь у 3 из них. Был отмечен рост уровня АТ к ТГ у 4 пациентов максимально до 566-кратного превышения относительно верхней границы референсного интервала. У одного пациента на протяжении 53 месяцев сохранялся стабильно 3-кратно повышенный уровень АТ к ТГ.

Были выявлены различия между пациентами в части накопления ¹³¹I метастатическим очагами: в одной группе отмечались признаки ¹³¹I-позитивной рефрактерности, при которой очаги накапливали ¹³¹I, но структурно не уменьшались. В этой подгруппе РЙТ была эффективнее (чаще наступала стабилизация/ремиссия), чем в исходно ¹³¹I-негативной группе (p<0,001) (табл. 6). Текущие данные дают основания для постановки гипотезы о необходимости выделения дополнительной группы «с неполным ответом на РЙТ», ввиду наличия слабоположительного ответа на РЙТ. В других случаях метастазы были ¹³¹I-негативными уже после первого курса, с сохранением структурных изменений по данным КТ в дальнейшем, и высоким уровнем ТГ.

**Table:** Таблица 6. Сравнение ¹³¹I-позитивных и ¹³¹I-негативных групп пациентов ¹ — критерий Манна-Уитни² — точный двусторонний критерий Фишера³ — критерий Фримена-ХолтонаПоправка Бонферрони: p0=0,05/7=0,007

Признак	¹³¹I-позитивные	¹³¹I -негативные	p
N	Me [ Q1; Q3]/ n (%)	N	Me [ Q1; Q3]/ n (%)
Суммарная активность ¹³¹I	23	14,7 [ 9,5; 22,9]	16	6,2 [ 4,1; 9,2]	<0,001¹
Исход заболевания	Стабилизация	23	17 (73,9%)	16	12 (75%)	0,134³
Ремиссия	4 (17,4%)	0 (0%)
Прогрессирование	2 (8,7%)	4 (25%)
Исход: стабилизация	23	17 (73,9%)	16	12 (75%)	0,946²
Исход: ремиссия	23	4 (17,4%)	16	0 (0%)	0,478²
Исход: прогрессирование	23	2 (8,7%)	16	4 (25%)	0,295²
Первый стимулированный ТГ до РЙТ	23	11,0 [ 0,4; 67,7]Min: 0Max: >5000	16	6,4 [ 1,0; 21,1]Min: 0Max: 347	0,577¹
Изменение ТГ	Снижение	23	17 (73,9%)	16	6 (37,5%)	0,579³
Без изменений	5 (21,7%)	9 (56,2%)
Повышение	1 (4,3%)	1 (6,3%)

В однофакторном анализе частота достижения ремиссии у пациентов с ¹³¹Iпозитивными метастазами оказалась выше: ремиссия была достигнута у 17,4% пациентов данной группы, тогда как в ¹³¹I-негативной группе ремиссия не была зарегистрирована ни у одного пациента. Тем не менее данное различие не достигло статистической значимости (p=0,478), что может быть связано с ограниченным объемом выборки.

При этом клинически прогрессирование заболевания также наблюдалось реже в ¹³¹I-позитивной группе — 8,7% против 25% во второй группе (p=0,295), без достижения статистической значимости, ввиду ограничения размера выборки.

Помимо различий в клинических исходах, между группами наблюдались выраженные отличия по параметрам, отражающим интенсивность лечения. Так, суммарная активность ¹³¹I, примененная в ходе терапии, была значительно выше в ¹³¹I-позитивной группе: медиана составила 14,7 ГБк [ 9,5; 22,9] против 6,2 ГБк [ 4,1; 9,2] во второй группе (p<0,001), что объясняется фактом накопления ¹³¹I в очагах, оправдывающим повторную терапию. Динамика уровня ТГ, являющегося маркером остаточной или рецидивной опухолевой ткани, также демонстрировала определенные тенденции. Несмотря на отсутствие статистически значимых различий (p=0,579), снижение или отсутствие роста, ТГ чаще отмечались в ¹³¹I-позитивной группе: у 73,9% пациентов зафиксировано снижение, у 21,7% — стабилизация уровня, тогда как в ¹³¹I-негативной группе пациентов эти показатели составили 37,5% и 56,2% соответственно.

Дополнительно можно отметить, что уровень ТГ после первой РЙТ также не отличался статистически значимо между группами (p=0,577), однако максимальные значения в ¹³¹I-позитивной группе достигали 21 310 нг/мл, в то время как в ¹³¹I-негативной лишь 347 нг/мл, что может быть связано с различной степенью дифференцировки опухоли, отражающейся на способности экспрессировать тиреоидные факторы, включая тиреоидспецифичные белки.

Для упрощения классификации в клинической практике мы разделили пациентов на подгруппы по динамике ответа на лечение и предлагаем следующую систему, учитывающую как начальный характер накопления ¹³¹I, так и динамику ответа на терапию. Эта система может служить основой для стратификации риска и выбора дальнейшей тактики лечения и наблюдения (рис. 2).

**Figure:**
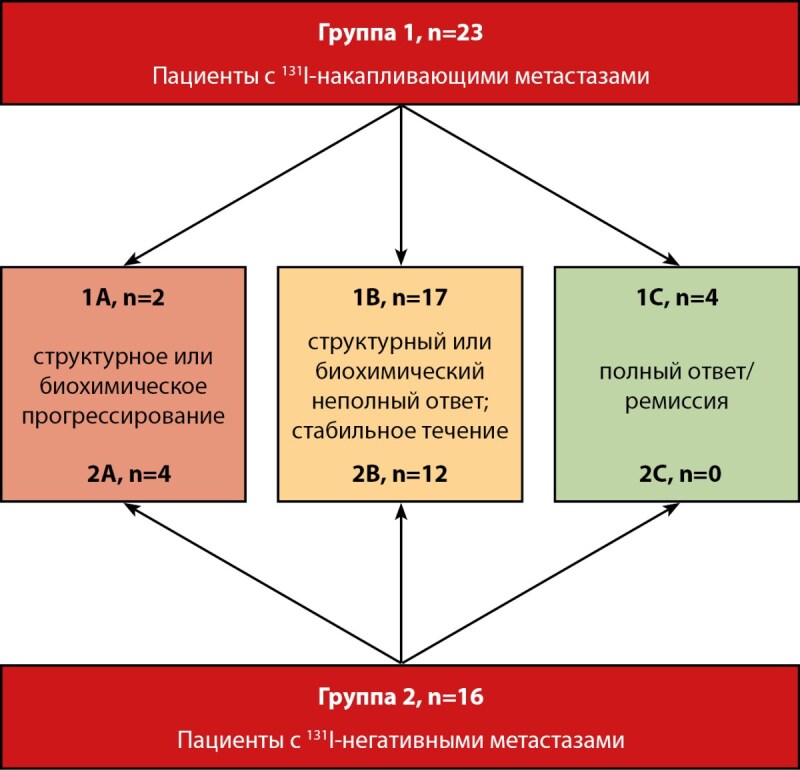
Рисунок 2. Классификация пациентов высокой группы риска на основании динамической стратификации, после комбинированного лечения. Группа 1 — пациенты с накоплением ¹³¹I в метастатических очагах (¹³¹I-позитивные); Группа 2 — пациенты без накопления ¹³¹I в метастатических очагах (¹³¹I-негативные); A, B, C (дополнительные индексы, присваиваемые каждой группе) — динамические показатели структурного и/или биохимического ответа

Предложенная классификация пациентов с РЙР ДРЩЖ, разделяющая их на группы по ответу на терапию, была клинически обоснована, но, вероятно, из-за небольшого объема выборки не получила статистически значимых различий. Полученные данные о более агрессивном течении заболевания с ¹³¹I-негативными метастазами коррелируют с данными международных исследований, что обосновывает их выделение в отдельную подгруппу [34–40]. Между исследуемыми группами были выявлены важные различия, отражающие различную клиническую динамику и ответ на терапию. Так, распределение исходов заболевания достоверно различалось между ¹³¹Iпозитивной и ¹³¹I-негативной группами (p=0,003), учитывая, что основные различия приходились на ремиссию и прогрессирование заболевания.

## Обсуждение

Основным показанием к проведению РЙТ у детей являются лечение отдаленных метастазов ДРЩЖ, а также — при определяемом уровне ТГ и/или АТ к ТГ — абляция остаточной ткани щитовидной железы после хирургического лечения с диагностической и лечебной целью. Абляция ¹³¹I остаточной, нормальной, тиреоидной ткани может способствовать ранней диагностике рецидива (на основании динамического определения уровня ТГ и АТ к ТГ в сыворотке крови и выполнения диагностической сцинтиграфии всего тела с радиойодом). При выявлении йод-накапливающих очагов опухоли большинство экспертов рекомендует проведение повторных курсов радиойодтерапии до полного отсутствия накопления ¹³¹I в очагах, их структурной элиминации, а также достижения неопределяемого уровня ТГ и/или АТ к ТГ. В случае ¹³¹I-негативных метастазов ДРЩЖ и невозможности проведения хирургического лечения, при выявлении прогрессирования заболевания в виде увеличения имеющихся очагов и/или появлении новых очагов может рассматриваться лекарственная терапия ТКИ. Объективную оценку ответа на лечение опухолевых очагов, как правило, проводят в процессе наблюдения согласно международной системе RECIST 1.1.

Определение тактики лечения должно осуществляться в специализированных центрах по решению консилиума мультидисциплинарной командой специалистов, имеющих опыт ведения данной категории больных.

В рамках настоящего исследования дети и подростки, у которых после одного или нескольких курсов РЙТ отсутствовали признаки патологического накопления ¹³¹I по данным птСВТ, но имелись лабораторные (повышение уровней ТГ и/или АТ к ТГ) и структурные (визуализируемые очаговые образования) признаки рецидива, были отнесены к категории резистентных к РЙТ, без морфологической верификации очагов ввиду инвазивности процедуры и тяжести ее выполнения в большинстве случаев.

Было отмечено, что пациенты с ¹³¹I-позитивными метастазами, которые в дальнейшем не достигли ремиссии и были классифицированы как РЙР, получили большую суммарную активность ¹³¹I. В нескольких крупных исследованиях РЙТ повышала риск вторичной онкологии на 50–60% в группе пациентов молодого возраста, хотя абсолютный риск данных событий остается низким [41][42]. При 20-летнем наблюдении 1 из 150 пациентов может приобрести вторичную опухолевую трансформацию органов риска. Вероятно, с течением лет процент вторичной малигнизации может увеличиться [41]. Несмотря на высокую кумулятивную активность, по данным зарубежных исследователей, полный ответ на терапию (не определяемый уровень ТГ и АТ к ТГ, отсутствие структурных изменений) будет наблюдаться лишь у части детей с распространенными формами заболевания (до 22%), при этом общий прогноз остается также благоприятным [43]. При отсутствии признаков быстрого прогрессирования метастатические очаги могут расцениваться как хроническое состояние, не требующее активного повторного лечения.

В данный момент алгоритм наблюдения пациентов детской возрастной группы, которым проведено комбинированное лечение по поводу ДРЩЖ, в своей сущности не отличается от такового у взрослых. Также и критерии резистентности к РЙТ у детей заимствованы из рекомендаций по лечению ДРЩЖ у взрослых.

Учитывая различную, чаще меньшую, чем у взрослых, активность ¹³¹I, используемую как с абляционной, так и с терапевтической целью, критерий суммарной терапевтической активности ¹³¹I должен быть пересмотрен в сторону уменьшения в детской когорте. При биохимическом рецидиве ДРЩЖ пациентам рекомендовано динамическое наблюдение с оценкой уровней ТГ и АТ к ТГ и времени их удвоения. При выявлении структурного рецидива на шее возможно проведение хирургического вмешательства при наличии показаний (отсутствии отдаленных метастазов/рисках инвазии органов шеи). В случае отдаленного метастазирования рекомендовано рассмотрение морфологической верификации выявленных образований с целью определения дальнейшей тактики, которая может заключаться в продолжении динамического наблюдения, назначении РЙТ или таргетной терапии. Распространение заболевания может быть косвенно подтверждено клинически, с учетом лабораторных маркеров, структурных (рентгенологических) характеристик образований, а также на основании накопления ¹⁸F-ФДГ при ПЭТ/КТ.

При внедрении предложенной классификации тактика ведения пациентов в группах будет различаться.

Группа 1 (¹³¹I -позитивные метастазы):

Группа 2 (¹³¹I -негативные метастазы):

Ограничения настоящего исследования:

## Заключение

Полученные данные во многом подтверждают международные: ДРЩЖ в педиатрической группе пациентов отличают распространенные формы заболевания [28][29][41] с более частым регионарным и отдаленным метастазированием, но при этом высокой общей выживаемостью пациентов. По некоторым данным, 30-летняя выживаемость пациентов с ДРЩЖ достигает 99% [44][45].

В настоящее время в ряде зарубежных работ демонстрируется, что РЙР ДРЩЖ встречается у 1/3 детей с отдаленным метастазированием и более распространен в возрасте до 15 лет [15][44], что может быть связано с доминированием онкогенных фьюжн-белков [45]. РЙР ДРЩЖ у детей может иметь различное течение в зависимости от исходной способности метастазов накапливать радиоактивный йод (¹³¹I-позитивные и ¹³¹I-негативные). В большинстве случаев заболевание протекает без прогрессирования в течение многих лет. Принимая во внимание повышенный риск возникновения вторичной онкологии, в частности хронического или острого миелобластного лейкоза и иных осложнений при повышении кумулятивной активности ¹³¹I, а также благоприятный профиль болезнь-специфической и безрецидивной выживаемости в длительной перспективе, можно прийти к следующим выводам:

На сегодняшний день, требуют обсуждения следующие аспекты ведения детей и подростков с ДРЩЖ: хирургическое лечение, терапия радиоактивным йодом (показания, активность ¹³¹I), критерии резистентности к РЙТ, периодичность и структура динамического наблюдения, назначение и коррекция терапии левотироксином натрия, а также таргетная терапия (показания, режимы и продолжительность).

Важно учитывать, что дети — не просто «уменьшенные копии» взрослых, а особая группа пациентов с уникальными характеристиками опухоли, молекулярными профилями и генетическими особенностями. Изучение вопросов патогенеза и тактики ведения ДРЩЖ у детей, в том числе резистентных к РЙТ, является актуальной современной проблемой.

На наш взгляд, существенным и необоснованным ограничением прогресса в изучении и совершенствовании тактики ведения РЙР ДРЩЖ у детей является критерий включения в клинические исследования пациентов старшее 18 лет. Во-первых, РЙР ДРЩЖ, как показывает наш опыт, не такое уж редкое явление. Во-вторых, это блокирует возможность применения современных противоопухолевых препаратов в детской группе. В‑третьих, терапевтический потенциал таргентных противоопухолевых препаратов, направленных на редифференцировку очагов опухоли и восстановление йод-накопительной способности, в детской когорте РЙР ДРЩЖ является еще более актуальным, чем во взрослой когорте.

Необходимо проведение проспективных мультицентровых исследований и создание единого регистра РЙР ДРЩЖ у детей в РФ, направленных на выработку более четких алгоритмов диагностики, лечения и наблюдения РЙР рака щитовидной железы. Возможными направлениями для поиска причин распространенного течения заболевания может являться изучение клональной гетерогенности опухоли между ¹³¹I-позитивными и ¹³¹I-негативными очагами, а также использование омиксных технологий, включая воксельную дозиметрию и углубленный молекулярный анализ. Эти данные помогут в выборе тактики ведения пациентов и принятии клинических решений между наблюдением или более активным лечебным вмешательством.

## Дополнительная информация

Информация о конфликте интересов. Авторы заявляют об отсутствии конфликта интересов.

Информация о финансировании. Работа по подготовке рукописи проведена в рамках государственного задания «Исследование фармакобезопасности тераностических радиофармацевтических лекарственных препаратов с использованием гибридной молекулярной визуализации в диагностике и лечении эндокринных и онкологических заболеваний в детской и взрослой возрастных группах». Регистрационный №ЕГИСУ НИОКТР 123021000041-6.

Вклад авторов. Все авторы одобрили финальную версию статьи перед публикацией, выразили согласие нести ответственность за все аспекты работы, подразумевающую надлежащее изучение и решение вопросов, связанных с точностью или добросовестностью любой части работы.
